# Multivariate analysis of prognostic factors in patients with lip squamous cell carcinoma after surgery

**DOI:** 10.1186/s12957-024-03313-9

**Published:** 2024-01-26

**Authors:** Hao Cheng, Jin-Hong Xu, Jia-Qi He, Xi-Yang Yang, Xu-Ning Shen, Xue-Lian Xu

**Affiliations:** 1 Department of Radiotherapy Oncology, The First Affiliated Hospital of Xinxiang Medical University, 88 Jiankang Road, Xinxiang, Henan 453100 China; 2 Department of Otolaryngology, Anyang District Hospital, Anyang, Henan 455000 China; 3 Department of Radiotherapy Oncology, Affiliated Cancer Hospital of Zhengzhou University, Zhengzhou, Henan 450000 China; 4 Department of Radiotherapy Oncology, Yuanyang County People’s Hospital, Xinxiang, Henan 453500 China

**Keywords:** Lip squamous cell carcinoma, Postoperative, Overall survival, Progression-free survival, Age-adjusted Charlson comorbidity index

## Abstract

**Background:**

Lip squamous cell carcinoma (LSCC) was one of the most common cancer types of head and neck tumors. This study aimed to find more predictors of the prognosis in postoperative LSCC patients.

**Methods:**

A total of 147 LSCC patients between June 2012 and June 2018 were collected from two tertiary care institutions. There were 21 clinicopathological factors included and analyzed in our study. The univariate and multivariate Cox regression analyses were performed to find the independent prognostic factors for predicting progression-free survival (PFS) and overall survival (OS) in postoperative LSCC patients. The role of adjuvant radiotherapy in various subgroups was displayed by Kaplan–Meier plots.

**Results:**

The 1-, 3-, and 5-year PFS of postoperative LSCC patients were 88.4%, 70.1%, and 57.8%, respectively. Similarly, the 1-, 3-, and 5-year OS of postoperative LSCC patients were 94.6%, 76.9%, and 69.4%, respectively. The results suggested that postoperative LSCC patients with age at diagnosis ≥ 70 years, grade with moderate or poor differentiate, the American Joint Committee on Cancer (AJCC) stage IV, higher systemic immune-inflammation index (SII), surgical margin < 5, and age-adjusted Charlson Comorbidity Index (ACCI) ≥ 5 tend to have a poorer PFS (all *P* < 0.05). Besides, postoperative LSCC patients with age at diagnosis ≥ 70 years, AJCC stage IV, higher GPS, higher SII, and *ACCI* ≥ 5 tend to have a worse OS (all *P* < 0.05). Additionally, postoperative patients with LSCC in the subgroup of *ACCI* < 5 and AJCC III–IV stage was more likely to benefit from adjuvant radiotherapy, but not for the other subgroups.

**Conclusion:**

We identified a series of significant immune-inflammation-related and comorbidity-related clinicopathological factors associated with the prognosis of postoperative LSCC patients by local data from two tertiary care institutions in China, which can be helpful for patients and surgeons to pay more attention to nutrition, inflammation, and complications and finally obtained a better prognosis.

**Supplementary Information:**

The online version contains supplementary material available at 10.1186/s12957-024-03313-9.

## Introduction

Lip squamous cell carcinoma (LSCC) is a type of oral cancer that accounts for about 25% of all oral cancer patients [1], and its incidence has shown a downward trend in recent years [2–4]. The lip was the junction of oral squamous cells and skin squamous cells, and LSCC was the pathological and anatomical combination of oral mucosa squamous cell carcinoma and skin squamous cell carcinoma. The aggressiveness and prognosis of LSCC were intermediate between those of oral mucosa squamous cell carcinoma and head and neck skin squamous cell carcinoma [5]. Smoking, long-term sunlight exposure, alcohol intake, and habitual chewing of betel nuts may be important acquired causes of lip cancer [6, 7]. Generally, the choice of treatment strategy and prognosis in LSCC patients depended on the traditional TNM AJCC stage. The tumor size, lymph node status, and distant metastasis are critical prognostic factors for those resected patients with LSCC, which has been reflected by the traditional TNM AJCC stage system. Surgery was still the main treatment for patients with LSCC [8, 9]. The prophylactic cervical lymph node dissection is the common method for estimating the lymph node status in LSCC patients. Besides, sentinel lymph node biopsy before surgical resection was another new method, which was mentioned in the notes of the Chinese Society of Clinical Oncology (CSCO) diagnosis and treatment guidelines for head and neck cancer (2018 version) [10] and has attracted the attention of many surgical experts, especially in patients with early-stage lip squamous cell carcinoma (LSCC) [11, 12]. However, due to the limitations of surgical techniques and other factors, the sentinel lymph node biopsy before surgical resection has not been commonly applied in all patients with early-stage lip squamous cell carcinoma (LSCC), especially in low-resource regions [13]. Recently, more and more clinicians have found that LSCC patients with the same TNM AJCC stage had an entirely different prognosis and therapeutic schedule in practice. There are some other factors that also affect the prognosis and treatment choice of LSCC patients, such as the patient’s general condition, immune status, nutritional status, and comorbidity. Therefore, it is important for us to find out more prognostic factors for LSCC patients, which can work as a complement to the traditional TNM AJCC stage system.

At present, there were risk factor analyses for the prognosis of patients with LSCC. These risk factors include age at diagnosis, marital status, sex, race, the American Joint Committee on Cancer (AJCC) stage, surgery status, positive lymph node ratio, total protein, immunoglobulin G, factor B, blood cell count, human papillomavirus (HPV) infection, and radiotherapy status [14–16]. However, there were few studies on the prognostic factors of postoperative LSCC patients. Therefore, a retrospective study was designed to find more predictors of the prognosis in postoperative LSCC patients, which can provide a certain reference for clinical application.

## Materials and methods

### Data collection

In this study, a total of 147 LSCC patients were obtained from the First Affiliated Hospital of Xinxiang Medical University and the Affiliated Cancer Hospital of Zhengzhou University between June 2012 and June 2018. The inclusion criteria were as follows: (1) pathology confirmed, (2) age at diagnosis ≥ 16, and (3) active follow-up. The exclusion criteria include the following: (1) There was distant metastasis at the time of initial diagnosis, (2) no radical resection was performed during the whole treatment, (3) patients who had received preoperative radiotherapy or preoperative chemotherapy, (4) dead within 30 days after surgery, (5) complete clinical data were not available, (6) patients who were lost to follow-up or did not cooperate with follow-up work, and (7) Eastern Cooperative Oncology Group performance status score (ECOG-PS) ≥ 3 before surgery.

We collected clinical data through the clinical case systems of the two medical institutions. A total of 21 clinicopathological variables of LSCC patients were included in the analysis, including age at diagnosis, gender, primary site, grade, AJCC stage, perineural invasion, vascular invasion, surgical margin, extranodal extension (ENE), adjuvant chemotherapy, adjuvant radiotherapy, ECOG-PS, Glasgow prognostic score (GPS), systemic immune-inflammation index(SII), prognostic nutrition index (PNI), platelet-to-lymphocyte ratio (PLR), age-adjusted Charlson Comorbidity Index (ACCI), neutrophil-to-lymphocyte ratio (NLR), hemoglobin, multiple primary tumors, and body mass index (BMI). The adjuvant chemotherapy in this study was platinum-based chemotherapy. The adjuvant radiotherapy was delivered by intensity-modulated radiotherapy (IMRT) with doses ranging from 50.0 to 71.0 Gy. A close margin was defined as a margin within 5 mm from the tumor. Overall survival (OS) and progression-free survival (PFS) were primary study endpoints.

### Calculations

ACCI is a marker implicated in comorbidity and age status. The calculation method for ACCI is shown in Table S1. Besides, BMI, GPS, PLR, PNI, NLR, and SII are indicators related to inflammation and nutrition, and the calculation formulas for those indexes are displayed in Table S2.

### Statistical analysis

All statistical analyses were performed by SPSS 20.0 software. Univariate Cox regression analysis was applied to find the potential predictors of OS and PFS. Then, these potential predictors were included for further analysis to reveal the independent predictors of OS and PFS by multivariate regression analysis. Additionally, all LSCC patients were divided into four subgroups based on the AJCC stage and ACCI to illustrate the benefit of adjuvant radiotherapy specifically (Table 4). Kaplan–Meier plots were used to display the benefit of adjuvant radiotherapy in these different subgroups. *P* < 0.05 was considered to be statistically significant.

## Results

### Patient clinicopathologic characteristics

A total of 147 LSCC patients were included in the study according to the inclusion criteria and exclusion criteria, with a median age of 66 years old. The 1-, 3-, and 5-year PFS of postoperative LSCC patients were 88.4%, 70.1%, and 57.8%, respectively. Similarly, the 1-, 3-, and 5-year OS of postoperative LSCC patients were 94.6%, 76.9%, and 69.4%, respectively. Of all cases, 112 patients (76.2%) with ECOG PS score of 0–1. The main primary site was lower lip (88.4%). Very few LSCC patients were with positive perineural invasion (8.2%) and vascular invasion (13.6%). Most patients were with negative ENE (87.8%) and had a surgical margin ≥ 5 (84.4%). There were 84 (57.1%) patients with LSCC who receive adjuvant radiotherapy after surgical resection.

There were a series of immune-inflammatory-nutritional indicators selected and analyzed in this study, and the indicators of SII (median 1138 (*IQR*: 686–1573)), PNI (median 73 (*IQR*: 49–96)), PLR (median 148 (*IQR*: 93–202)), and NLR (median 2.42 (*IQR*: 1.34–3.29)) were analyzed as continuous variables. The GPS was analyzed as categorical variable, which was divided into three groups according to the score of C-reactive protein and albumin, 103 (70.1%) patients with 0 score, 24 (16.3%) patients with 1 score, and 20 (13.6%) patients with 2 score. Moreover, hemoglobin (HGB) is also a marker implicated in nutritional status, which was also included in analysis with a median of 101 g/l (*IQR*: 90 g/l–121 g/l). Finally, 87 (59.2%) LSCC patients were with an ACCI score of < 5, and 60 (40.8%) LSCC patients were with an ACCI score of ≥ 5. All baseline of clinicopathological characteristics was summarized in Table 1.

**Table 1:** Clinicopathological characteristics of postoperative LSCC patients

Characteristics	Value	Characteristics	Value
No. of patients	147	ECOG PS	
Age at diagnosis (years)		0–1	112 (76.2%)
< 70	92 (62.6%)	2	35 (23.8%)
≥ 70	55 (37.4%)	GPS	
Gender		0	103 (70.1%)
Male	71 (48.3%)	1	24 (16.3%)
Female	76 (51.7%)	2	20 (13.6%)
Primary site		SII	
Upper lip	17 (11.6%)	Median	1138
Lower lip	130 (88.4%)	IQR	686–1573
Grade		PNI	
Well differentiate	66 (44.9%)	Median	73
Moderate differentiate	44 (29.9%)	IQR	49–96
Poor differentiate	37 (25.2%)	PLR	
AJCC stage		Median	148
I	28 (19.0%)	IQR	93–202
II	39 (26.5%)	ACCI	
III	52 (35.4%)	< 5	87 (59.2%)
IV	28 (19.0%)	≥ 5	60 (40.8%)
Perineural invasion		NLR	
No	135 (91.8%)	Median	2.42
Yes	12 (8.2%)	IQR	1.34–3.29
Vascular invasion		Hemoglobin (g/L)	
No	127 (86.4%)	Median	101
Yes	20 (13.6%)	IQR	90–121
Surgical margin (mm)		Multiple primary tumors	
< 5	23 (15.7%)	Yes	11 (7.5%)
≥ 5	124 (84.4%)	No	136 (92.5%)
ENE		BMI (kg/m^2^)	
Negative	129 (87.8%)	Median	21.3
Positive	18 (12.2%)	IQR	19.6–25.0
Adjuvant chemotherapy		OS (months)	
No	94 (63.9%)	Median	72
Yes	53 (36.1%)	Range	6–133
Adjuvant radiotherapy		PFS (months)	
No	63 (42.9%)	Median	64
Yes	84 (57.1%)	Range	4–132

Abbreviations: *ACCI* age-adjusted Charlson comorbidity index, *AJCC* American Joint Committee on Cancer, *BMI* body mass index, *ECOG-PS*, Eastern Cooperative Oncology Group performance status score, *ENE* extranodal extension, *GPS* Glasgow prognostic score, *IQR* inter-quartile range, *LSCC*, lip squamous cell carcinoma, *NLR* neutrophil-to-lymphocyte ratio, *OS* overall survival, *PFS* progress-free survival, *PLR* platelet-to-lymphocyte ratio, *PNI* prognostic nutrition index, *SII* systemic immune-inflammation index

There were 11 patients with multiple primary tumors: two patients had lung cancer, two had basal cell carcinoma, three had thyroid cancer, three had malignant lymphoma, and one had both oropharyngeal and breast cancer.

### Univariate and multivariate Cox regression results

The clinicopathological factors associated with OS and PFS of postoperative LSCC patients were identified by the univariate and multivariate Cox regression analyses. Univariate Cox regression analysis showed that age at diagnosis, grade, AJCC stage, GPS, SII, PNI, ACCI, vascular invasion, surgical margin, and ENE were significant factors for PFS (Table 2). Besides, nine variables (age at diagnosis, AJCC stage, GPS, SII, PNI, ACCI, vascular invasion, surgical margin, and ENE) were found to be associated with OS (Table 3). Consequently, independent prognostic factors associated with PFS and OS were figured out by multivariate Cox regression analysis respectively. The independent predictors related to PFS were as follows: age at diagnosis [≥ 70 years: hazard ratio (HR) = 2.986 (95% confidential interval (CI) = 1.780–5.009), *P* = 0.001], grade [moderate differentiate: *HR* = 1.895 (95% *CI* = 1.078–3.332), *P* = 0.026; poor differentiate: *HR* = 2.688 (95% *CI* = 1.447–4.995), *P* = 0.002], AJCC stage [IV: *HR* = 3.814 (95% *CI* = 1.682–8.651), *P* = 0.001], SII [*HR* = 1.000 (95% *CI* = 1.000–1.001), *P* = 0.013], ACCI [≥ 5: *HR* = 1.731 (95% *CI* = 1.075–2.788), *P* = 0.024], surgical margin [≥ 5: *HR* = 0.523 (95% *CI* = 0.282–0.970), *P* = 0.04]. The independent prognostic factors related to OS include the following: age at diagnosis [≥ 70: *HR* = 2.334 (95% *CI* = 1.382–3.976), *P* = 0.002], AJCC stage [IV: *HR* = 3.841 (95% *CI* = 1.516–9.734), *P* = 0.005], GPS [1: *HR* = 1.986 (95% *CI* = 1.020–3.864), *P* = 0.043; 2: *HR* = 2.127 (95% *CI* = 1.070–4.229), *P* = 0.003], SII [*HR* = 1.000 (95% *CI* = 1.000–1.001), *P* = 0.003], ACCI [≥ 5: *HR* = 2.402 (95% *CI* = 1.403–4.111), *P* = 0.001]. The detailed result was shown in Tables 2 and 3.

**Table 2:** Univariate and multivariate analyses results for PFS in postoperative LSCC patients

Characteristics	Univariate analysis		Multivariate analysis	
	**HR (95% *CI*)**	***P***	**HR (95% *CI*)**	***P***
Age at diagnosis (years)				
< 70	Reference		Reference	
≥ 70	2.294 (1.522–3.458)	< 0.001***	2.986 (1.780–5.009)	0.001***
Gender				
Male	Reference			
Female	0.862 (0.553–1.343)	0.511		
Primary site				
Upper	Reference			
Lower	1.051 (0.541–2.041)	0.884		
Grade		0.081		
Well differentiate	Reference			
Moderate differentiate	1.541 (0.908–2.616)	0.109	1.895 (1.078–3.332)	0.026*
Poor differentiate	1.819 (1.053–3.141)	0.032*	2.688 (1.447–4.995)	0.002**
Perineural invasion				
No	Reference			
Yes	1.641 (0.789–3.413)	0.185		
AJCC stage				
I	Reference		Reference	
II	1.824 (0.843–3.946)	0.127	1.219 (0.548–2.714)	0.628
III	2.855 (1.369–5.957)	0.005**	2.196 (1.001–4.819)	0.050
IV	5.393 (2.443–11.906)	< 0.001***	3.814 (1.682–8.651)	0.001***
BMI	0.962 (0.909–1.019)	0.189		
Hemoglobin	0.994 (0.983–1.004)	0.207		
ECOG PS				
0–1	Reference			
2	1.066 (0.641–1.771)	0.806		
GPS				
0	Reference		Reference	
1	1.536 (0.874–2.700)	0.136	1.823 (0.998–3.332)	0.051
2	2.302 (1.242–4.265)	0.008**	1.654 (0.876–3.123)	0.121
SII	1.000 (1.000–1.000)	0.003**	1.000 (1.000–1.001)	0.013*
PNI	0.986 (0.976–0.995)	0.003**	0.989 (0.979–1.000)	0.047
PLR	1.001 (0.998–1.003)	0.518		
NLR	1.081 (0.890–1.312)	0.433		
ACCI				
< 5	Reference		Reference	
≥ 5	2.108 (1.343–3.308)	0.001***	1.731 (1.075–2.788)	0.024*
Vascular invasion				
No	Reference		Reference	
Yes	2.233 (1.268–3.934)	0.005**	0.918 (0.473–1.782)	0.800
Surgical margin (mm)				
< 5	Reference		Reference	
≥ 5	0.530 (0.306–0.919)	0.024*	0.523 (0.282–0.970)	0.040*
ENE				
Negative	Reference		Reference	
Positive	2.312 (1.291–4.139)	0.005**	1.055 (0.533–2.088)	0.878
Multiple primary tumors				
No	Reference			
Yes	2.125 (1.017–4.440)	0.045		
Adjuvant chemotherapy				
No	Reference			
Yes	0.704 (0.438–1.132)	0.147		
Adjuvant radiotherapy				
No	Reference			
Yes	0.752 (0.484–1.170)	0.205		

Abbreviations: *ACCI* age-adjusted Charlson comorbidity index, *AJCC* American Joint Committee on Cancer, *BMI* body mass index, *CI* confidential interval, *ECOG-PS* Eastern Cooperative Oncology Group performance status score, *ENE* extranodal extension, *GPS* Glasgow prognostic score, *HR* hazard ratio, *LSCC* lip squamous cell carcinoma, *NLR* neutrophil-to-lymphocyte ratio, *PFS* progression-free survival, *PLR* platelet-to-lymphocyte ratio, *PNI* prognostic nutrition index, *SII* systemic immune-inflammation index

*P < 0.05

**P < 0.01

***P < 0.001

**Table 3:** Univariate and multivariate analyses results for OS in postoperative LSCC patients

Characteristics	Univariate analysis		Multivariate analysis	
	**HR (95% *CI*)**	***P***	**HR (95% *CI*)**	***P***
Age at diagnosis (years)				
< 70	Reference		Reference	
≥ 70	2.351 (1.452–3.805)	0.001***	2.334 (1.382–3.976)	0.002**
Gender				
Male	Reference			
Female	0.738 (0.456–1.197)	0.218		
Primary site				
Upper	Reference			
Lower	1.566 (0.676–3.626)	0.295		
Grade		0.177		
Well differentiate	Reference			
Moderate differentiate	1.356 (0.760–2.419)	0.302		
Poor differentiate	1.742 (0.969–3.133)	0.064		
Perineural invasion				
No	Reference			
Yes	1.758 (0.802–3.856)	0.159		
AJCC stage				
I	Reference		Reference	
II	1.942 (0.817–4.615)	0.133	1.240 (0.507–3.036)	0.637
III	3.497 (1.554–7.868)	0.002**	2.150 (0.888–5.207)	0.090
IV	5.963 (2.458–14.464)	< 0.001***	3.841 (1.516–9.734)	0.005**
BMI	0.976 (0.918–1.037)	0.428		
HGB	0.994 (0.983–1.005)	0.283		
ECOG PS				
0–1	Reference			
2	0.981 (0.559–1.722)	0.946		
GPS				
0	Reference		Reference	
1	1.539 (0.824–2.874)	0.176	1.986 (1.020–3.864)	0.043*
2	2.892 (1.533–5.453)	0.001***	2.127 (1.070–4.229)	0.031*
SII	1.000 (1.000–1.001)	< 0.001***	1.000 (1.000–1.001)	0.003**
PNI	0.985 (0.975–0.995)	0.004**	0.992 (0.980–1.003)	0.146
PLR	1.002 (0.999–1.004)	0.167		
NLR	1.139 (0.920–1.410)	0.231		
ACCI				
< 5	Reference		Reference	
≥ 5	2.597 (1.587–4.252)	< 0.001***	2.402 (1.403–4.111)	0.001***
Vascular invasion				
No	Reference		Reference	
Yes	2.390 (1.342–4.257)	0.003**	0.953 (0.489–1.858)	0.887
Surgical margin (mm)				
< 5	Reference		Reference	
≥ 5	0.477 (0.272–0.837)	0.010**	0.521 (0.276–0.984)	0.045
ENE				
Negative	Reference		Reference	
Positive	2.703 (1.495–4.889)	0.001***	1.854 (0.936–3.674)	0.077
Multiple primary tumors				
No	Reference			
Yes	1.990 (0.856–4.627)	0.110		
Adjuvant chemotherapy				
No	Reference			
Yes	0.824 (0.497–1.364)	0.452		
Adjuvant radiotherapy				
No	Reference			
Yes	0.808 (0.500–1.306)	0.385		

Abbreviations: *ACCI* age-adjusted Charlson comorbidity index, *AJCC* American Joint Committee on Cancer, *BMI* body mass index, *CI* confidential interval, *ECOG-PS* Eastern Cooperative Oncology Group performance status score, *ENE* extranodal extension, *GPS* Glasgow prognostic score, *HR* hazard ratio, *LSCC* lip squamous cell carcinoma, *NLR* neutrophil-to-lymphocyte ratio, *OS* overall survival, *PLR* platelet-to-lymphocyte ratio, *PNI* prognostic nutrition index, *SII* systemic immune-inflammation index

*P < 0.05

**P < 0.01

***P < 0.001

**Fig. 1:**
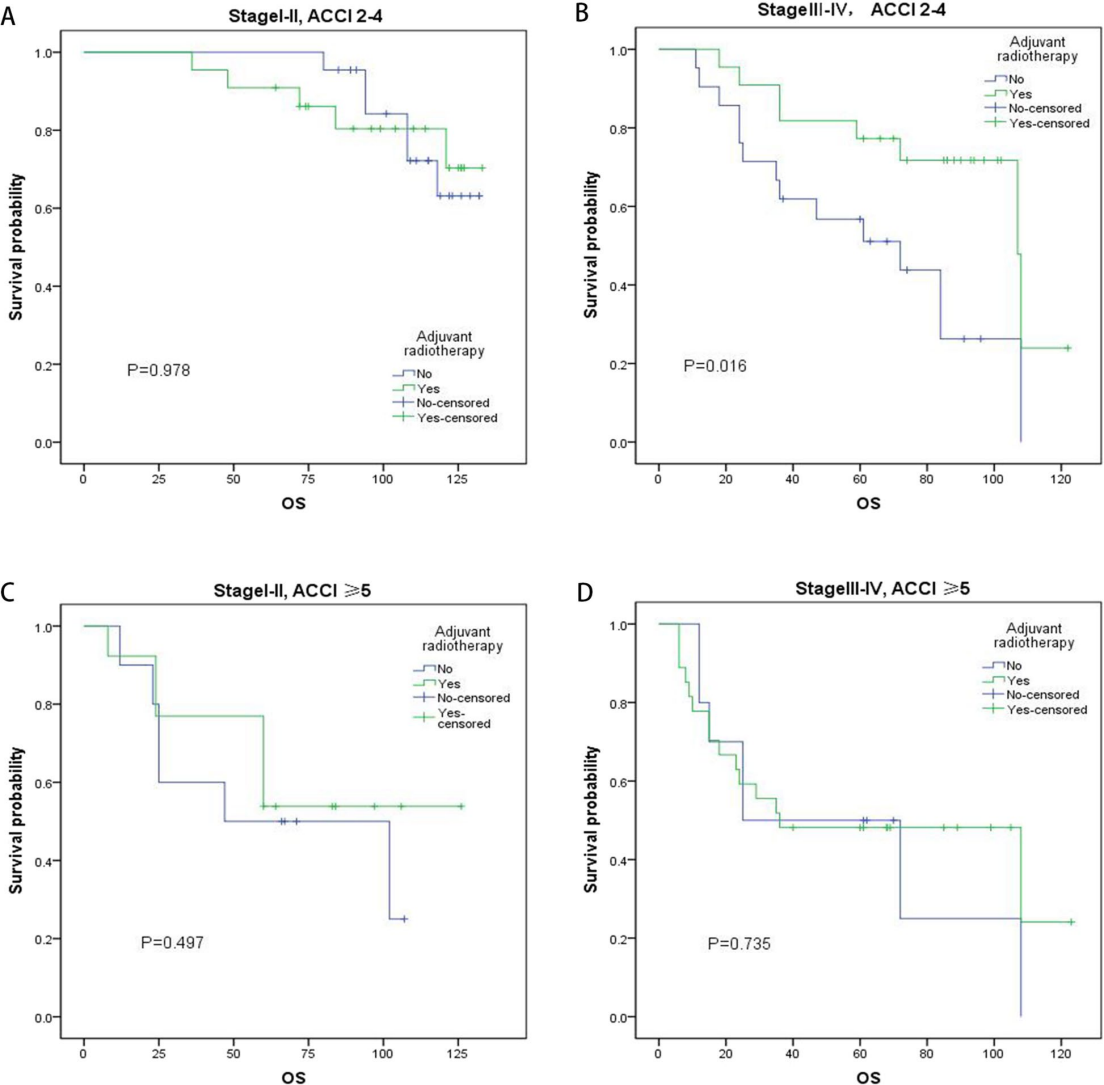
The effects of adjuvant radiotherapy on OS in different subgroups by Kaplan–Meier curves. **A** AJCC stages I–II and ACCI 2–4. **B** AJCC stages III–IV and ACCI 2–4. **C** AJCC stages I–II and *ACCI* ≥ 5. **D** AJCC stages III–IV and *ACCI* ≥ 5. ACCI, age-adjusted Charlson Comorbidity Index; OS, overall survival

**Fig. 2:**
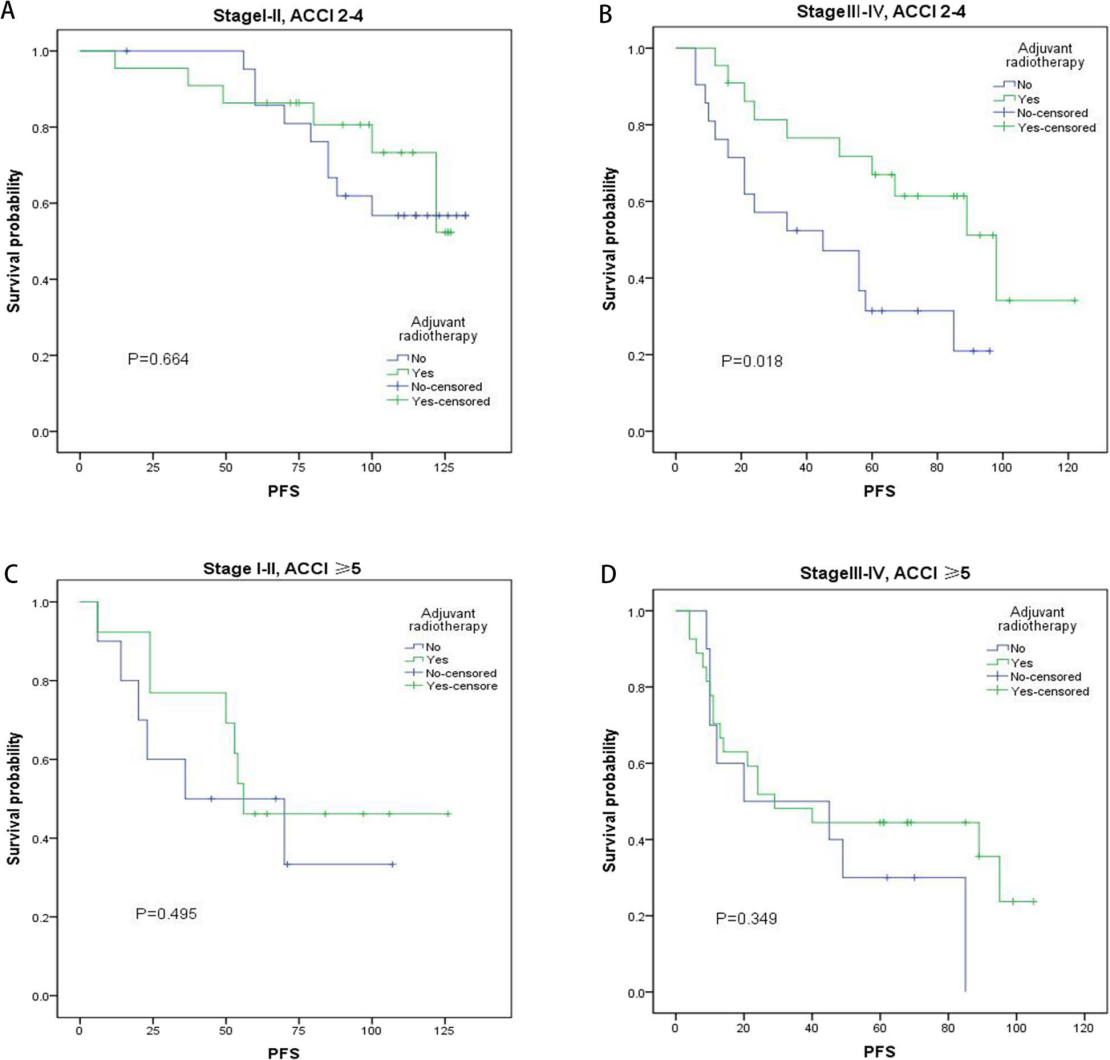
The effects of adjuvant radiotherapy on PFS in different subgroups by Kaplan–Meier curves. **A** AJCC stages I–II and ACCI 2–4. **B** AJCC stages III–IV and ACCI 2–4. **C** AJCC stages I–II and *ACCI* ≥ 5. **D** AJCC stages III–IV and *ACCI* ≥ 5. ACCI, age-adjusted Charlson comorbidity index; PFS, progression-free survival

Additionally, all enrolled postoperative patients with LSCC were divided into four subgroups according to the ACCI and AJCC stage, and the benefits of adjuvant radiotherapy on OS and PFS were displayed by the Kaplan–Meier plots in Table 4, Figs. 1 and 2. Obviously, there was a considerable benefit of prognosis in the subgroup of *ACCI* < 5 and AJCC stages III–IV for LSCC patients who receive adjuvant radiotherapy, but not for the other subgroups.

**Table 4:** Impact of adjuvant radiotherapy on PFS and OS in different subgroups of postoperative LSCC patients

**Subgroups**	Adjuvant radiotherapy	**PFS**		**OS**	
		**Chi-square**	***P***	**Chi-square**	***P***
AJCC stages I–II & *ACCI* < 5 (*n* = 44)	No (*n* = 22)	0.188	0.664	0.001	0.978
	Yes (*n* = 22)				
AJCC stages III–IV & *ACCI* < 5 (*n* = 43)	No (*n* = 21)	5.846	0.018*	5.598	0.016*
	Yes (*n* = 22)				
AJCC stages I–II & *ACCI* ≥ 5 (*n* = 23)	No (*n* = 10)	0.465	0.495	0.461	0.497
	Yes (*n* = 13)				
AJCC stages III–IV & *ACCI* ≥ 5 (*n* = 37)	No (*n* = 10)	0.878	0.349	0.114	0.735
	Yes (*n* = 27)				

Abbreviations: *ACCI* age-adjusted Charlson comorbidity index, *AJCC* American Joint Committee on Cancer, *LSCC* lip squamous cell carcinoma, *PFS* progression-free survival, *OS* overall survival

*P < 0.05

## Discussion

LSCC was a common type of oral cancer. Mostly, the AJCC stage system was commonly applied to predict the prognosis of LSCC patients [1, 17]. Previously, various studies indicate that local advanced stage LSCC patients tend to have a poorer prognosis [18, 19], which is consistent with our study. However, increasing studies suggest that other clinicopathological factors also had an important effect on the prognosis of LSCC patients except for the traditional AJCC stage system.

Age at diagnosis is a commonly used factor in assessing the general condition of cancer patients. There was a different morbidity and mortality in different age groups for most cancers. Wolfer S. et al. [20] showed that age at diagnosis had a different effect on prognosis depending on gender in oral cancer patients. However, some researchers suggest that age has little effect on prognosis or has an effect only in certain populations [21, 22]. It was controversial in the impact of age on the prognosis of LSCC patients. In this study, older patients with LSCC have shorter survival times, which was similar to the results of previous studies [14, 18, 23].

Tumor differentiation is a traditional clinical factor, which commonly have an important impact on the prognosis of patients with cancer to some extent. It has been always taken into account in the evaluation of prognosis and treatment choice in various cancers. Vesna Janevska et al. [24] found that poorer differentiation can increase the density of neoangiogenesis in lower LSCC patients. In this study, we found that LSCC patients with moderate or poor differentiate tend to have a relatively worse PFS in comparison to LSCC patients with well differentiate, which is consistent with previous studies. As is well known, OS is more likely to be affected by multiple clinical or social factors than PFS. Therefore, it is reasonable that tumor differentiation is not an important factor for predicting OS of LSCC patients according to our results.

Although the definition of “close margin” has always been a controversial topic in oral cancer surgery, there is a consensus that close margin may improve local recurrence rate and reduce long-term survival [25–28]. Our study suggested that a surgical margin distance within 5 mm from the tumor is unsafe and will predict a poor prognosis. Due to the delicate anatomical structure of the maxillofacial region, excessive surgical margins may destroy organ function and cause cosmetic defects. Further studies with a larger sample size were needed to determine the appropriate surgical margins.

Previous researchers had paid their attention to ENE and vascular invasion in oral cancer [3, 17–20]. In our study, although ENE and vascular invasion were not the independent prognostic factors, they had significantly negative effects on both OS and PFS in postoperative LSCC patients by univariate Cox analysis. If the pathological and imaging results show ENE and/or vascular invasion, the clinicians should adjust the treatment intensity and follow-up frequency.

As we all know, inflammation-related indicators generally play an important role in tumor microenvironment and treatment [29–32]. Complete blood count (FBC), C-reactive protein (CRP), and albumin can reflect the systemic inflammatory status of cancer patients and thus predict prognosis to some extent [29, 33]. SII, GPS, and PNI are indicators of systemic inflammation status calculated from the above clinical data, reflecting not only systemic inflammation but also the antitumor response and immune monitoring status of the patient. Increased neutrophil counts or neutrophilia in cancer occur due to the secretion of myeloid growth factors by tumor cells triggering neutrophil production or due to cancer-related inflammation secondary to tissue destruction or hypercytokinemia [34]. Lymphocytopenia and neutropenia are often associated with reduced antitumor response and immunosuppression [29, 35–37]. Numerous studies have revealed a negative correlation between SII and tumor prognosis [38–42]. Both GPS and PNI incorporate serum albumin levels, which reflect a patient’s nutritional, immune, and inflammatory status, and were all important prognostic factors [43–47]. However, the effects of SII, PNI, and GPS on the prognosis of LSCC had not been studied. In our study, higher SII was independent predictors of PFS and OS. Besides, the higher the GPS, the worse the OS in LSCC patients according to the results. PNI has some effects on the prognosis of LSCC patients, but was not the independent predictors of prognosis. All these results were an important supplement to the study of LSCC.

The ACCI is a useful comorbidity indicator, which is commonly utilized to normalize the assessment of patients of different ages and has been reported to predict the mortality of various cancers [48–50]. In our study, for LSCC patients who underwent adjuvant radiotherapy, the OS (*P* = 0.016) and PFS (*P* = 0.018) were significantly improved in subgroup of AJCC stages III–IV and *ACCI* < 5. Moreover, in the subgroup of *ACCI* ≥ 5 or AJCC stages I–II, adjuvant radiotherapy did not have any benefit in improving the prognosis. Therefore, it is importantly that we should not only refer to the AJCC stage system but also pay more attention to patients’ ACCI scores in choosing the optimal treatment strategy for postoperative LSCC patients.

There are also some limitations in our study. Firstly, this was a retrospective study, which had some inevitable selective bias. Secondly, due to the low incidence of LSCC, the selected cases were small and cannot reflect the situation of all LSCC patients in China. Finally, some potential prognostic factors were not included in the analysis, such as pain score, tumor markers, dietary habits, marital status, and preoperative mean platelet volume. Further research should be conducted to find more prognostic factors in LSCC patients.

## Conclusion

In summary, a series of significant immune-inflammation-related and comorbidity-related clinicopathological factors associated with the prognosis of postoperative LSCC patients were identified in this study. It is helpful for patients and surgeons to pay more attention to nutrition, inflammation, and complications and finally obtained a better prognosis.

### Supplementary Information

**Additional file 1: Table S1.** Age-adjusted Charlson Comorbidity Index. **Table S2.** Calculation formulas in this study.

## Data Availability

Detailed data are available from the corresponding author on reasonable request.

## Abbreviations

ACCI: Age-adjusted Charlson comorbidity index
AJCC: American Joint Committee on Cancer
BMI: Body mass index
CI: Confidential interval
CSCO: Chinese Society of Clinical Oncology
ECOG-PS: Eastern Cooperative Oncology Group performance status score
ENE: Extranodal extension
GPS: Glasgow prognostic score
HPV: Human papillomavirus
HR: Hazard ratio
IMRT: Intensity-modulated radiotherapy
IQR: Inter-quartile range
LSCC: Lip squamous cell carcinoma
NLR: Neutrophil-to-lymphocyte ratio
OS: Overall survival
PFS: Progression-free survival
PLR: Platelet-to-lymphocyte ratio
PNI: Prognostic nutrition index
SII: Systemic immune-inflammation index
