# Supplementary material for: Multivariate analysis of prognostic factors in patients with lip squamous cell carcinoma after surgery
Source: World J Surg Oncol. 2024 Jan 26;22:35. doi: 10.1186/s12957-024-03313-9 (PMC10811904; doi:10.1186/s12957-024-03313-9)
Supplement: Supplementary file 1 — Additional file 1: Table S1. Age-adjusted Charlson Comorbidity Index. Table S2. Calculation formulas in this study. [file 12957_2024_3313_MOESM1_ESM.zip › Table S1.docx]

| **Table S1**. Age-adjusted Charlson Comorbidity Index. | |
| --- | --- |
| **Clinical conditions included in the score** | **Scores** |
|  | **Scores for Disease** |
| Myocardial infarction; Peripheral vascular disease; Congestive heart failure; Dementia; Chronic pulmonary disease; Connective tissue disease; Diabetes; Cerebrovascular disease; Ulcer disease; Mild liver disease | 1 point |
| Hemiplegia; Diabetes with endo organ damage; Moderate or severe renal disease; Any tumor; Leukemia; Lymphoma | 2 points |
| Moderate or severe liver disease | 3 points |
| Metastatic solid tumor; Acquired immune deficiency syndrome | 6 points |
|  | **Scores for Age** |
| Each decade over age 40 years (up to 4 points) | 1 point |
