# Supplementary material for: Multivariate analysis of prognostic factors in patients with lip squamous cell carcinoma after surgery
Source: World J Surg Oncol. 2024 Jan 26;22:35. doi: 10.1186/s12957-024-03313-9 (PMC10811904; doi:10.1186/s12957-024-03313-9)
Supplement: Supplementary file 1 — Additional file 1: Table S1. Age-adjusted Charlson Comorbidity Index. Table S2. Calculation formulas in this study. [file 12957_2024_3313_MOESM1_ESM.zip › Table S2.docx]

| **Table S2**. Calculation formulas in this study. | |
| --- | --- |
| **Clinical Index** | **Calculation Formula** |
| SII | Platelet count × (Neutrophil count / lymphocyte count) |
| GPS | Score of 0 if C-reactive protein ≤ 10 mg/dL and albumin ≥ 35 g/L  Score of 1 if C-reactive protein > 10 mg/dL or albumin < 35 g/L  Score of 2 if C-reactive protein > 10 mg/dL and albumin < 35 g/L |
| PNI | Serum albumin (g/L) + (lymphocyte count × 5) |
| NLR | Neutrophil count / lymphocyte count |
| PLR | Platelet count / lymphocyte count |
| BMI | Mass (kg) / height^2^(m^2^) |
| Abbreviations: BMI, body mass index; GPS, Glasgow prognostic score; PLR, platelet-to-lymphocyte ratio; PNI, prognostic nutrition index; NLR, neutrophil-to-lymphocyte ratio; SII, systemic immune-inflammation index. | |
